# Compare rates of deaths and injuries of the accident occurred on the roads in Turkey between the years 2012-2017

**Published:** 2019-08

**Authors:** Hakan Gulmez, Zeynep Sofuoglu

**Affiliations:** ^*a*^Izmir Democracy University, Faculty of Medicine, Turkey.

## Abstract

**Background:**

The dictionary definition of safety is “The condition of being protected from or unlikely to cause danger, risk, or injury.”1 Safety is a fundamental human right. In addition, the dictionary definition of road safety is “teaching people how to behave safely when driving or crossing the road.”2 The UN High Commissioner for Human Rights said: “Road safety is a human rights issue. Today, international law brings positive obligations to every State to take all reasonable steps to protect the right to life, the right to personal security, the right to health and the right to develop all persons. Every year, many vehicles are involved in accidents on highways. Road traffic accidents are leading cause of death and disability globally. The aim of this study is to investigate the road traffic accidents occurred in Turkey between the years 2012-2017 and to compare the rates of deaths and injuries by vehicle type involved in these accidents.

**Methods:**

Road traffic accidents occurred in Turkey between the years 2012-2017 constitute the universe of the study. The study data was taken from the Turkish Statistical Institute. Percentages were calculated with Microsoft office 365 ProPlus Exel. Percentage distributions were used to compare the values.

**Results:**

The total number of vehicles involved in accidents between 2012 and 2017 was 240 thousand, 251 thousand, 264 thousand, 290 thousand, 295 thousand and 294 thousand respectively. Tractor accidents were the most fatal accidents with 8-10% death rate. Tractor accidents were followed by tractor trailer accidents with a 5-8% death rate. Truck accidents were the third deadly vehicle type with a 4-7% death rate. Contrary to expectations, motorcycle accidents had the lowest mortality rate (0.8-2%), but there is no finding about the permanent disabilities and DALY. Cars were the second in the ranking of safe vehicles according to mortal accident rates.

**Conclusions:**

The data shows there was no difference in fatality or injury rates by years.

**Keywords:**

Road safety, Fatality rates, Injury rates, Accident

